# Enhanced disease resistance and drought tolerance in transgenic rice plants overexpressing protein elicitors from *Magnaporthe oryzae*

**DOI:** 10.1371/journal.pone.0175734

**Published:** 2017-04-18

**Authors:** Zhenzhen Wang, Qiang Han, Qian Zi, Shun Lv, Dewen Qiu, Hongmei Zeng

**Affiliations:** The State Key Laboratory for Biology of Plant Diseases and Insect Pests, Institute of Plant Protection, Chinese Academy of Agricultural Sciences, Beijing, China; Institute of Genetics and Developmental Biology Chinese Academy of Sciences, CHINA

## Abstract

Exogenous application of the protein elicitors MoHrip1 and MoHrip2, which were isolated from the pathogenic fungus *Magnaporthe oryzae* (*M*. *oryzae*), was previously shown to induce a hypersensitive response in tobacco and to enhance resistance to rice blast. In this work, we successfully transformed rice with the *mohrip1* and *mohrip2* genes separately. The *MoHrip1* and *MoHrip2* transgenic rice plants displayed higher resistance to rice blast and stronger tolerance to drought stress than wild-type (WT) rice and the vector-control *pCXUN* rice. The expression of salicylic acid (SA)- and abscisic acid (ABA)-related genes was also increased, suggesting that these two elicitors may trigger SA signaling to protect the rice from damage during pathogen infection and regulate the ABA content to increase drought tolerance in transgenic rice. Trypan blue staining indicated that expressing MoHrip1 and MoHrip2 in rice plants inhibited hyphal growth of the rice blast fungus. Relative water content (RWC), water usage efficiency (WUE) and water loss rate (WLR) were measured to confirm the high capacity for water retention in transgenic rice. The *MoHrip1* and *MoHrip2* transgenic rice also exhibited enhanced agronomic traits such as increased plant height and tiller number.

## Introduction

*Magnaporthe oryzae (M*. *oryzae)* is a plant-pathogenic fungus that causes rice blast, a destructive rice plant disease, around the world [1]. Traditional fungicides are usually used to control blast; however, this approach gradually increases the potential resistance of *M*. *oryzae* to fungicides and has adverse effects on human health and the environment [2,3]. Rice plants face biotic and abiotic stress in nature that can cause severe losses in production. The transgenic approach is an effective strategy for generating disease-resistant, drought-resistant and high-quality rice varieties.

The effectors produced by plant pathogens during plant-pathogen interactions have been isolated from plant pathogenic bacteria, oomycetes, and fungi [4–6]. The broader definition of effectors includes avirulence factors, elicitors, pathogen-associated molecular patterns (PAMPs), toxins, and degradative enzymes [7]. Effectors improve plant defenses against pathogens and activate defense pathways to induce systemic acquired resistance (SAR) in plants [8]. With the development of genetic transformation technology, numerous examples exist of enhanced pathogen resistance through the transgenic expression of protein elicitors: The harpin-encoding gene has been expressed successfully in transgenic tobacco and rice, showing pathogen resistance [8–10]; flagellin expression induces disease resistance in transgenic rice [11]; constitutive expression of the PemG1 gene from *M*. *oryzae* in transgenic rice results in enhanced resistance to the rice blast fungus [12]; and transgenic *Arabidopsis* plants expressing the Hrip1 elicitor show resistance to abiotic and biotic stressors such as salt, drought, and *Botrytis cinerea* [13].

MoHrip1 and MoHrip2 are two secreted protein elicitors isolated from the rice blast fungus *M*. *oryzae*. The *mohrip1* gene (GenBank accession No. JQ231215.1) consists of 429 bp encoding 142 amino acids, and the *mohrip2* gene (GenBank accession No. JQ815555.1) consists of 459 bp encoding 152 amino acids. Moreover, the exogenous application of these elicitors in rice seedlings induces the early events of the defense response in tobacco, such as reactive oxygen species (ROS) and enhanced resistance to the blast fungus *M*. o*ryzae* [14,15].

In this article, we investigated the expression and function of the *mohrip1* and *mohrip2* genes in rice. Transgenic rice plants constitutively expressing MoHrip1 and MoHrip2 were generated. Our results demonstrated that *MoHrip1* and *MoHrip2* transgenic rice plants display enhanced resistance to *M*. *oryzae* and tolerance to drought stress.

## Materials and methods

### Construction of the expression vector and the transformation of rice

**Fig 1:**
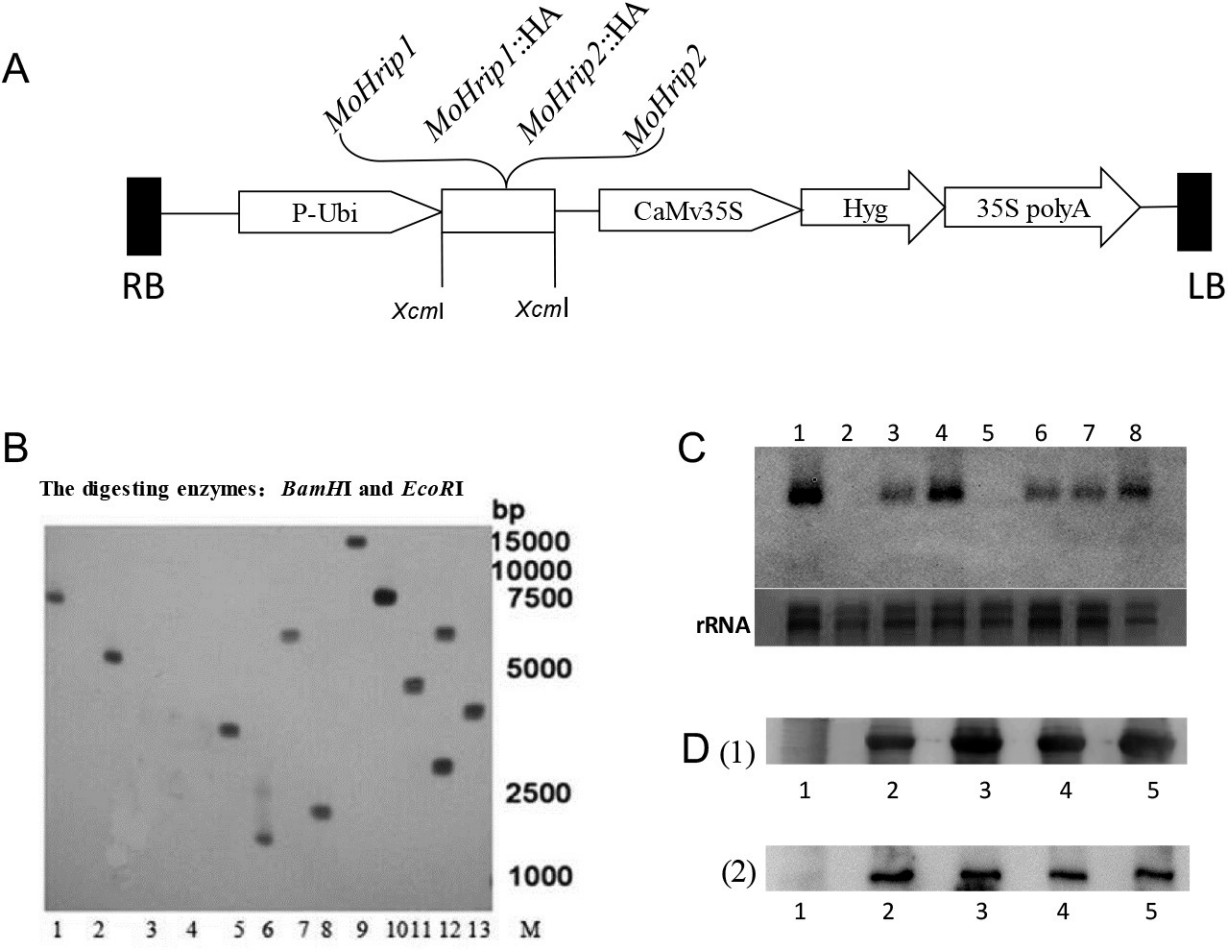
The integration and expression of MoHrip1 and MoHrip2 in rice. Different transgenic rice lines were chosen for molecular detection. (A). Schematic representation of the T-DNA region of pCXUN containing MoHrip1/MoHrip2-encoding genes. (B). Southern blotting of T_1_ transgenic rice. The numbers (1–13) represent *MoHrip1*-5, *MoHrip1*-9, *MoHrip1*-1, *MoHrip1*::HA-2, *MoHrip1*::HA-5, *MoHrip1*::HA-3, *MoHrip1*::HA-1, *MoHrip2*-5, *MoHrip2*-8, *MoHrip2*- 4, *MoHrip2*::HA-8, *MoHrip2*::HA-4, and *MoHrip2*::HA-4, respectively. M: DL15000 Marker. (C). Northern blotting of T_2_ transgenic rice. The numbers (1–8) represent *MoHrip1*-5, *MoHrip1*::HA-3, *MoHrip1*::HA-5, *MoHrip1*::HA-1, *MoHrip2*-8, *MoHrip2*-5, *MoHrip2*::HA-4, and *MoHrip2*::HA -8, respectively. The loading controls were 18S RNA and 28S rRNA. (D). Western blotting of the T_2_ transgenic rice. *pCXUN* was used as a negative control. (d1) The numbers (1–3) represent the empty vector *pCXUN*, *MoHrip1*::HA-1, *MoHrip1*::HA-5, respectively, and numbers 4 and 5 represent *MoHrip1*-5; (d2) The numbers (1–3) represent *pCXUN*, *MoHrip2*::HA -4, *MoHrip2*::HA -8, respectively, and the numbers 4 and 5 represent *MoHrip2*-5. All samples verified the expression of MoHrip1 and MoHrip2 in transgenic rice.

The *mohrip1* and *mohrip2* gene regions without a signal peptide were amplified using polymerase chain reaction (PCR), digested with *Xcm*I, and an HA-tag was fused to the C-terminus of the coding sequences. The fragments were cloned into pCXUN under the control of a maize ubiquitin promoter [16]. Four different expression constructs (*MoHrip1*, *MoHrip1*::HA, *MoHrip2*, and *MoHrip2*::HA) and the vector control pCXUN were separately cloned under the control of the maize ubiquitin promoter (Fig 1A). Four recombinant plasmids and the vector control pCXUN were used to separately transform *Agrobacterium tumefaciens* strain LBA4404. *Agrobacterium-*mediated rice transformation was performed as described previously [17]. The PCR primers used are listed in Table 1 (P1-P8).

**Table 1:** Primers used in this study.

Primer	Sequence	DNA
**P1**	5’-atggcccctgccccgcaggcgcaggccacctcg-3’	*MoHrip1*-F
**P2**	5’-ctaagcggagccgtcaatggcaatagcggc-3’	*MoHrip1*-R1
**P3**	5’-atgcagcaggctatcgtccacaacaactgccag-3’	*MoHrip2*-F
**P4**	5’-ctaggcgcagagggtagcctcgacgttgatgg-3’	*MoHrip2*-R1
**P5**	5’-ctaagcgtaatctggaacatcgtatgggtaagcggagccgtcaatggcaatag-3’	*MoHrip1*::HA-R
**P6**	5’-ctaagcgtaatctggaacatcgtatgggtaggcgcagagggtagcctcgacgttgatgg-3’	*MoHrip2*::HA-R
**P7**	5’-agcggagccgtcaatggcaatagcggc-3’	*MoHrip1*-R2
**P8**	5’-ggcgcagagggtagcctcgacgttgatgg-3’	*MoHrip2*-R2

The HA sequence is underlined

### Cultivation of transgenic rice

The rice cultivar Nipponbare (*Oryza sativa* spp. *japonica*) was used in this study. The T_0_ generation seeds were obtained by selecting embryonic calli for hygromycin resistance [17]. Then, both T_1_ generation and T_2_ generation breeding was conducted at the breeding base of the Fujian Academy of Agricultural Sciences in Sanya. Transgenic plants were cultivated for research in a greenhouse as described previously [14]. The experiments were performed in triplicate with 25 seedlings from each line under the same environmental conditions.

*MoHrip1*::HA and *MoHrip2*::HA transgenic rice and vector-control *pCXUN* rice were selected to test agronomic traits at the breeding base. Before mowing, both the plant height and thousand-kernel weight were measured; the tiller number, ear number and days from sowing to mowing were then recorded. Seeds were germinated on moistened filter paper placed in Petri dishes. The germination rates were counted with 100 seeds from each line. Three days after germination, the bud length was measured, and then the seedlings were transferred to hydroponic cultures. After 20 days, the root length was recorded.

### PCR and Southern blot analysis

Genomic DNA was isolated from the tender leaves of T_0_, T_1_ and T_2_ rice seedlings using the Cetyltrimethylammonium bromide (CTAB) method [18]. The primer pairs P1/P7 and P3/P8 were designed to amplify *mohrip1* and *mohrip2*, respectively, from transgenic rice (Table 1). Genomic DNA was digested using *BamH*Ι and *EcoR*Ι, separated via 1.2% agarose gel electrophoresis, and then transferred onto Hybond-N nylon membranes (Amersham, Uppsala, Sweden) for Southern blotting [19]. The MoHrip1 and MoHrip2 probes used in the experiment were labeled using the Dig-High primer method. Hybridization was performed according to the Dig-High Primer DNA Labeling and Detection Starter Kit I (Roche, Germany).

### Real-time (RT)-PCR and Northern hybridization

Leaf samples for RNA extraction were placed in liquid nitrogen, and the TRIzol reagent (Invitrogen, Carlsbad, CA, USA) was used according to the manufacturer’s instructions. For RT-PCR, first-strand cDNA was prepared using the FastQuant RT Kit (Transgen, Beijing, China) under the following conditions: 95°C for 3 min, 27 cycles of 94°C for 30 s, 62°C for 30 s and 72°C for 45 s, followed by 72°C for 10 min.

For Northern hybridization, total RNA was separated on 1.2% agarose gel containing formaldehyde and transferred onto nylon membranes. The probes were the same as those used for Southern blotting. Hybridization was performed as described previously (Roche, Germany).

### Protein extraction and Western blotting

Crude protein was extracted from T_2_ positive transgenic rice, as confirmed via Northern hybridization, using a precooled extraction buffer (0.025 mol L^-1^K_2_HPO_4_, 0.025 mol L^-1^KH_2_PO_4_, and 2 mmol L^-1^EDTA, p H 8.0). The protein suspension was concentrated and then separated via 12% sodium dodecyl sulfate polyacrylamide gel electrophoresis (SDS-PAGE) for Western blotting.

Polyclonal antibodies against MoHrip1 or MoHrip2 were produced in New Zealand white rabbits caged in the laboratory, and goat anti-rabbit antibody (Transgen, Beijing, China) was used as the secondary antibody. The separated proteins were transferred to a PVDF membrane via semi-dry electroblotting. The membrane was blocked with 5% BSA in TBST (10 mM Tris–HCl, 100 mM NaCl, 0.2% Tween-20) and incubated for 12 h at 4°C with antibody in TBST containing 5% BSA (1:2,000 dilution) and then alkaline phosphatase-tagged goat anti-rabbit IgG in TBST (1:2,000 dilution).

### Quantitative (q) RT-PCR

For qRT-PCR, a FastQuant RT kit and SYBR Green (Tiangen) were used with a CFX96 real-time PCR machine (Bio-Rad Laboratories, Hercules, CA, USA). The thermal cycling conditions were as follows: denaturation at 95°C for 15 s, followed by 40 cycles of 95°C for 10 s, and 60°C for 30 s. Nipponbare Actin (GenBank accession No. AK060893) was amplified as an internal control. Each reaction was performed in triplicate. The qRT-PCR primers used are listed in S1 Table.

### Inoculation of rice with *M*. *oryzae* spores

*M*. *oryzae* (KJ201) was used in the inoculation experiments. To evaluate the resistance of transgenic rice plants to rice blast infection, detached leaves were inoculated with blast suspension spores [20]. The second leaves of 2-week-old rice plants, 3 lines of *MoHrip1* rice (*MoHrip1*-5 *MoHrip1*::HA -1, and *MoHrip1*::HA -5), 3 lines of *MoHrip2* rice *(MoHrip2*-5, *MoHrip2*::HA -4, and *MoHrip2*::HA -8), wild-type (WT) rice, and *pCXUN* rice, were placed onto different dishes with 1% water agar containing 2 mg L^-1^ kinetin. Whatman filter paper discs saturated with *M*. *oryzae* KJ201 suspension spores at a concentration of 2 ×10^5^ spores per ml were placed onto the upper face of the leaf for 48 h in the dark. The inoculated leaves were then maintained at 28°C with 90% relative humidity under a 14-h light/10-h dark photoperiod. Leaves were then stained with Trypan blue solution at 2 and 6 days post-inoculation (dpi) [21].

Soil-grown plants were also sprayed with *M*. *oryzae* spores. Four transgenic rice plants, *MoHrip1*, *MoHrip1*::HA, *MoHrip2*, and *MoHrip2*::HA, were tested for resistance to rice blast, whereas *pCXUN* rice and WT rice were used as controls. At the three-leaf stage, plants were sprayed with blast suspension spores at a concentration of 2×10^5^ per ml containing 0.05% Tween20. The inoculated plants were placed in a dark chamber for 24 h at 25°C with 100% relative humidity and then transferred to a growth chamber at 28°C with 90% relative humidity under 14-h light/10-h dark conditions. The disease severity of the transgenic plants was recorded at 7 and 15 dpi. A scale of 0 to 9 was used [14], where 0 = no visible symptoms and 9 = maximum development. Leaves were collected from *MoHrip1*, *MoHrip2*, *pCXUN*, and WT rice for qRT-PCR.

### Drought stress tolerance assays of seedlings

*MoHrip1*-5 and *MoHrip2*-5 transgenic plants as well as *pCXUN* rice and WT rice were well-watered for 14 d, and then the plants were subjected to a non-irrigation period until the soil moisture content decreased to 6%, as measured by oven drying and weighing [22].

On the 0 d, 6 d, 10 d, 14 d, and 16 d of drought stress, leaves were collected from *MoHrip1*, *MoHrip2*, *pCXUN* and WT rice across three biological replicates to analyze gene expression and detect ABA content. ABA was quantified by high performance liquid chromatography–mass spectrometry (HPLC-MS) using an UltiMate 3000 HPLC system (Thermo Scientific, Dionex, Sunnyvale, California, USA) and a Q Exactive mass spectrometer (Thermo Scientific, Bremen, Germany). The method was performed according to previous studies [23].

### Measurement of physiological indices under the drought stress

The water loss rate (WLR) was measured under dehydration conditions [24]. Three-leaf stage seedlings detached from roots were exposed to air at room temperature. The plants were weighed at 0, 0.5, 2, 4, 6, 8, 12, and 24 h after being cut off. The plants were finally oven dried for 48 h at 80°C to a constant dry weight (DW). WLR was measured according to the following formula: WLR (%) = (FW–desiccated weight) / FW × 100. The relative water content (RWC) was measured according to a previously described method [25]. The desiccated weight of the dehydrated leaves was recorded, and then the leaves were soaked in distilled water at room temperature. After 4 h, the turgid weight (TW) was recorded. The leaves were finally dried for 24 h at 80°C to obtain the total dry weight (DW). RWC was calculated using the following formula: RWC (%) = [(desiccated weight–DW) / (TW–DW)] × 100. Photosynthetic parameters were analyzed using an open gas-exchange system (LI-6400; Li-Cor, Lincoln, NE) [26]. We used an LI-6400 portable photosynthesis analyzer equipped with blue and red light sources to measure photosynthesis using a 1000 μmol m−2 s−1 PPFD and 500 μmol s−1 flow rate. Photosynthesis parameters were analyzed between 9:00 and 11:00 h in outdoor conditions using the second leaf. Water usage efficiency (WUE) was calculated as follows: WUE = (net photosynthetic rate) / (transpiration rate). Each measurement was performed three times in independent trials, using four replicates for each treatment. Relative chlorophyll content was measured using a portable TYS-A chlorophyll meter (Top Instrument, Zhejiang, China).

### Statistical analysis

All experiments and data provided in this paper were repeated three times. Statistical analysis was carried out in both Microsoft Excel and Statistical Analyses System for Windows V8 (SAS, NC State, USA). The data are presented as the means ± the standard deviation. Variance analysis was conducted by comparing the statistical difference based on Student’s t-test (*P < 0.05, **P < 0.01).

## Results

### The generation and molecular characterization of transgenic rice

The following T_0_ transgenic plants were generated: 102 *MoHrip1*, 47 *MoHrip1*::HA, 63 *MoHrip2*, 101 *MoHrip2*::HA, and 71 vector control *pCXUN*. The single-copy rate of T_1_ transgenic plants reached 75%, and the transgenic plants segregated at 3:1 for hygromycin resistance (S2 Table). PCR results showed the *mohrip1* or *mohrip2* was present in the genomic DNA of T_0_, T_1_ and T_2_ plants (results not shown). Southern blotting showed one or two transgene insertions determined the integration patterns of the transgenic rice (Fig 1B). Northern hybridization revealed the expression of the *mohrip1* or *mohrip2* gene in the T_2_ transgenic plants (Fig 1C). The results of Western blotting indicated that the crude protein of T_2_ transgenic rice showed a positive reaction at the expected sizes of MoHrip1 or MoHrip2 (Fig 1D).

### MoHrip1 or MoHrip2 expression enhances resistance to rice blast and triggers host defense responses

**Fig 2:**
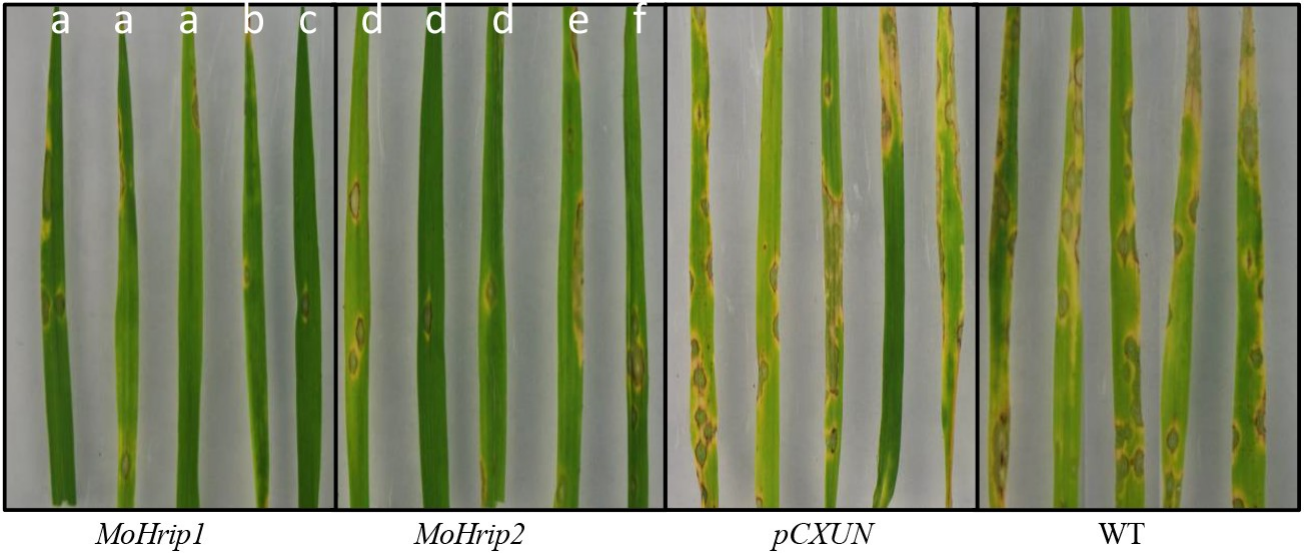
Disease symptoms on the leaves of *MoHrip1*, *MoHrip2*, *pCXUN*, and WT rice. The detached leaves of two-week-old rice seedlings were sprayed with *M*. *oryzae* spores. The lowercase letters (a-f) represent *MoHrip1*-5 *MoHrip1*::HA-1, *MoHrip1*::HA-5 *MoHrip2*-5, *MoHrip2*::HA -4, and *MoHrip2*::HA -8, respectively. Representative leaves were photographed at 7 dpi. The results were obtained from three independent experiments.

The colonization by blast fungus in *MoHrip1* and *MoHrip2* rice plants was determined using detached leaves. The leaves from 3 lines of the *MoHrip1* and *MoHrip2* plants were assayed, and WT and *pCXUN* rice were used as controls. At 3 dpi, no lesions were observed on the leaves of the *MoHrip1* and *MoHrip2* plants; however, symptoms were observed on the leaves of control plants. At 7 dpi, typical and severe rice blast symptoms were observed on the leaves of control plants, and the sizes of the lesions obviously increased; in contrast, the lesions on the leaves of the *MoHrip1* and *MoHrip2* plants were small and constrained, and almost all leaves on the plate remained green. The lesion sizes on *MoHrip1*, *MoHrip2*, *pCXUN*, and WT rice leaves were ~1.41%, ~1.71%, ~8.63%, and ~9.56%, respectively. Moreover, slightly fewer lesions occurred on the leaves of the *MoHrip1* rice than those on the leaves of the *MoHrip2* rice (Fig 2).

**Fig 3:**
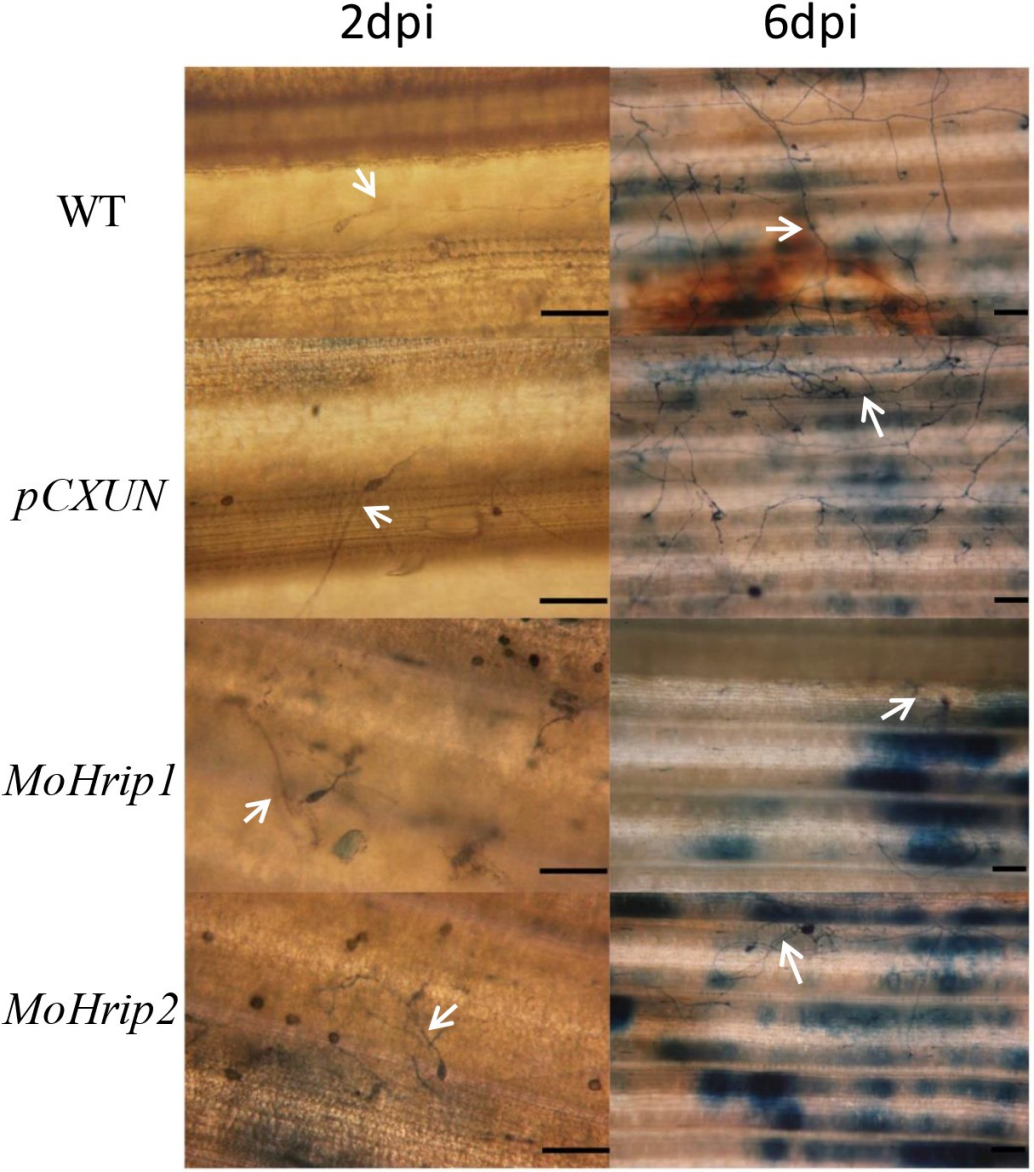
The Trypan blue staining of leaves challenged with *M*. *oryzae* spores. The 2- and 6-dpi leaves were stained with Trypan blue solution to observe the development of disease symptoms at the inoculation sites. The arrows indicate the hyphae (2 dpi: bars = 100 μm; 6 dpi: bars = 50 μm).

Trypan blue staining was conducted to confirm the fungal development results at the inoculation sites. Under the microscope, the hyphae within the control plants were long and normally developed, whereas the hyphae within the *MoHrip1* and *MoHrip2* plants were short and constricted at 2 dpi. Many hyphae were observed on the leaves of control plants, whereas coiled and abnormal hyphal growth was observed only on the leaves of the *MoHrip1* and *MoHrip2* plants at 6 dpi (Fig 3). Fig 3 shows slightly fewer hyphae on the *MoHrip1* rice than on the *MoHrip2* rice.

The disease severity of the WT and transgenic plants was evaluated on a scale of 0–9 at 7 and 15 dpi. The leaf blast scores of the *MoHrip1* and *MoHrip2* rice lines were much lower than that of the control plants, and the *MoHrip1* lines showed slightly lower levels of disease severity than the *MoHrip2* lines (Table 2). Most of the control plants wilted at 15 dpi and showed retarded growth. By this time, the *MoHrip1* and *MoHrip2* plants continued to grow well and showed fewer leaf lesions, and the disease severity of the *MoHrip1* rice was slightly lower than that of the *MoHrip2* rice (date not shown).

**Table 2:** Disease severity of rice blast in leaves of transgenic rice.

Rice seedling samples		Rice blast score
	7 days post- inoculation	15 days post- inoculation
***MoHrip1***	3.24±0.56 A	3.30±0.43 A
***MoHrip1*::HA**	3.38±0.77 A	3.44±0.53 A
***MoHrip2***	3.47±0.48 A	3.65±0.34 A
***MoHrip2*::HA**	3.83±0.49 A	4.20±0.55 A
***pCXUN***	5.62±0.43 B	6.25±0.58 B

Soil-grown rice seedlings were sprayed with *M*. *oryzae* spores. The disease scores of the rice seedlings were evaluated on a scale of 0–9 at 7 and 15 dpi. The data represent three replicates and twenty plants per replicate. Values represent means ± standard deviations. Within columns, values with different letters are significantly different at P < 0.01.

**Fig 4:**
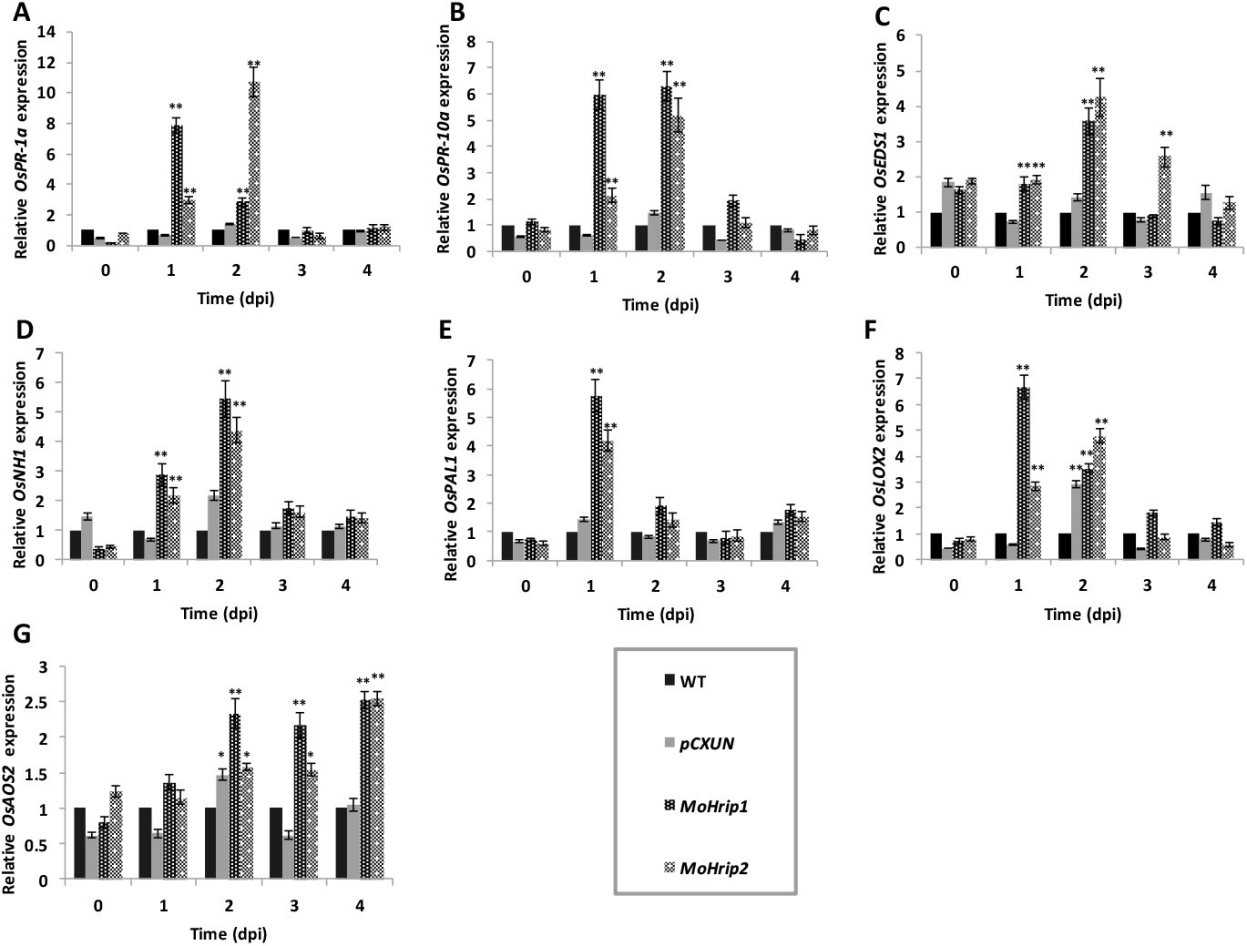
The expression levels of defense-related genes in rice. The WT, *pCXUN*, *MoHrip1* and *MoHrip2* rice were inoculated with blast suspension spores (2×10^5^). At the indicated times, leaves were collected from these rice plants and measured via qRT-PCR. The relative expression of pathogenesis-related genes *OsPR-1a* (A) and *OsPR-10a* (B); SA signal-related genes *OsEDS1* (C), *OsNH1* (D), and *OsPAL1* (E); and JA/ET biosynthesis-related genes *OsLOX2* (F) and *OsAOS2* (G) are shown. dpi: days post-inoculation; Error bars represent mean ± SD. Essentially identical results were obtained across three independent experiments. The asterisks indicate significant differences from the WT rice (*P < 0.05, **P < 0.01)

Resistance to blast in transgenic rice was also determined by analyzing the expression of defense-related genes: two pathogenesis-related genes (*OsPR-1a* and *OsPR-10a*), three SA signal-related genes (*OsEDS1*, *OsNH1*, and *OsPAL1*) and two JA/Et biosynthesis-related genes (*OsLOX2* and *OsAOS2*) were selected (Fig 4A–4G). *OsPR-1a* and *OsPR-10a* expression was obviously induced in the *MoHrip1* and *MoHrip2* transgenic rice at 1 and 2 dpi. The expression of *OsPR-1a* reached its maximum level at 1 dpi in the *MoHrip1* rice and at 2 dpi in the *MoHrip2* rice (Fig 4A). The expression level of *OsPR-10a* was rapidly induced in the *MoHrip1* rice at 1 dpi and continued through 2 dpi, whereas it was induced in the *MoHrip2* rice at 2 dpi (Fig 4B). The expression of the signal-related genes *OsEDS1*, *OsNH1*, *OsPAL1*, and *OsLOX2* reached maximum levels at 1 or 2 dpi and was markedly induced in the *MoHrip1* and *MoHrip2* rice (Fig 4C–4F). Moreover, the expression of the *OsAOS2* gene increased gradually within 5 days and had no similar pattern to the other genes (Fig 4G).

### MoHrip1 or MoHrip2 expression increased drought stress tolerance in rice

**Fig 5:**
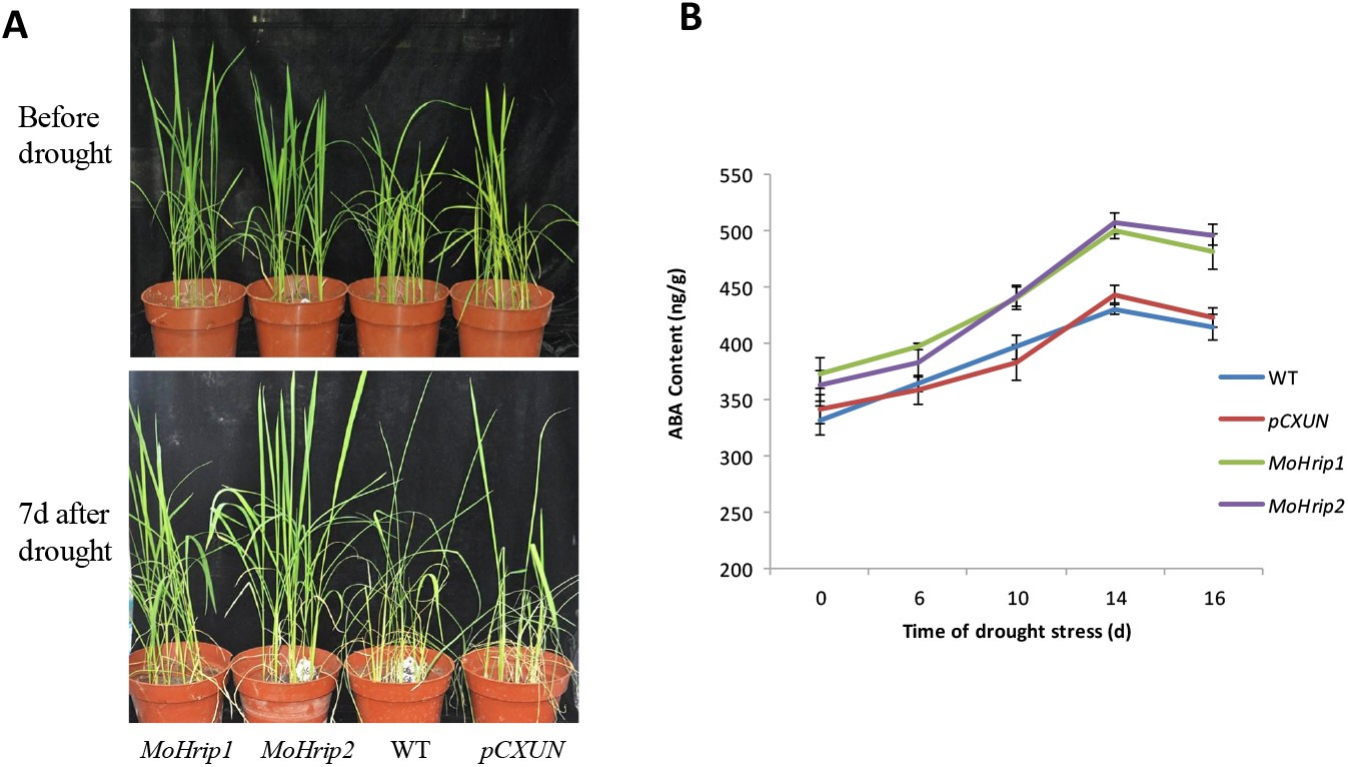
The phenotype and ABA content of drought-stressed rice. The *MoHrip1*-5 and *MoHrip2*-5 transgenic plants (line 5 of each transgenic plant) as well as the *pCXUN* rice and WT rice were well-watered for 2 weeks, and then water was discontinued for 16 days. The phenotype of rice before drought and after drought (A). The ABA content of transgenic and control rice on the 0 d, 6 d, 10 d, 14 d, and 16 d of drought stress (B). Essentially identical results were obtained across three independent experiments.

The *MoHrip1*-5 and *MoHrip2*-5 transgenic plants, as well as the *pCXUN* and WT rice, were well-watered for 2 weeks, and then water was discontinued. After 7 days, most of the control rice showed wilting, although half of the transgenic rice remained upright (Fig 5A). Drought stress led to an increase in the ABA content of the transgenic and control rice. Compared to the *pCXUN* and WT rice, the *MoHrip1* and *MoHrip2* transgenic rice maintained relatively higher ABA content. Moreover, the differences in ABA content between the transgenic and control rice were significant under drought stress (Fig 5B).

**Fig 6:**
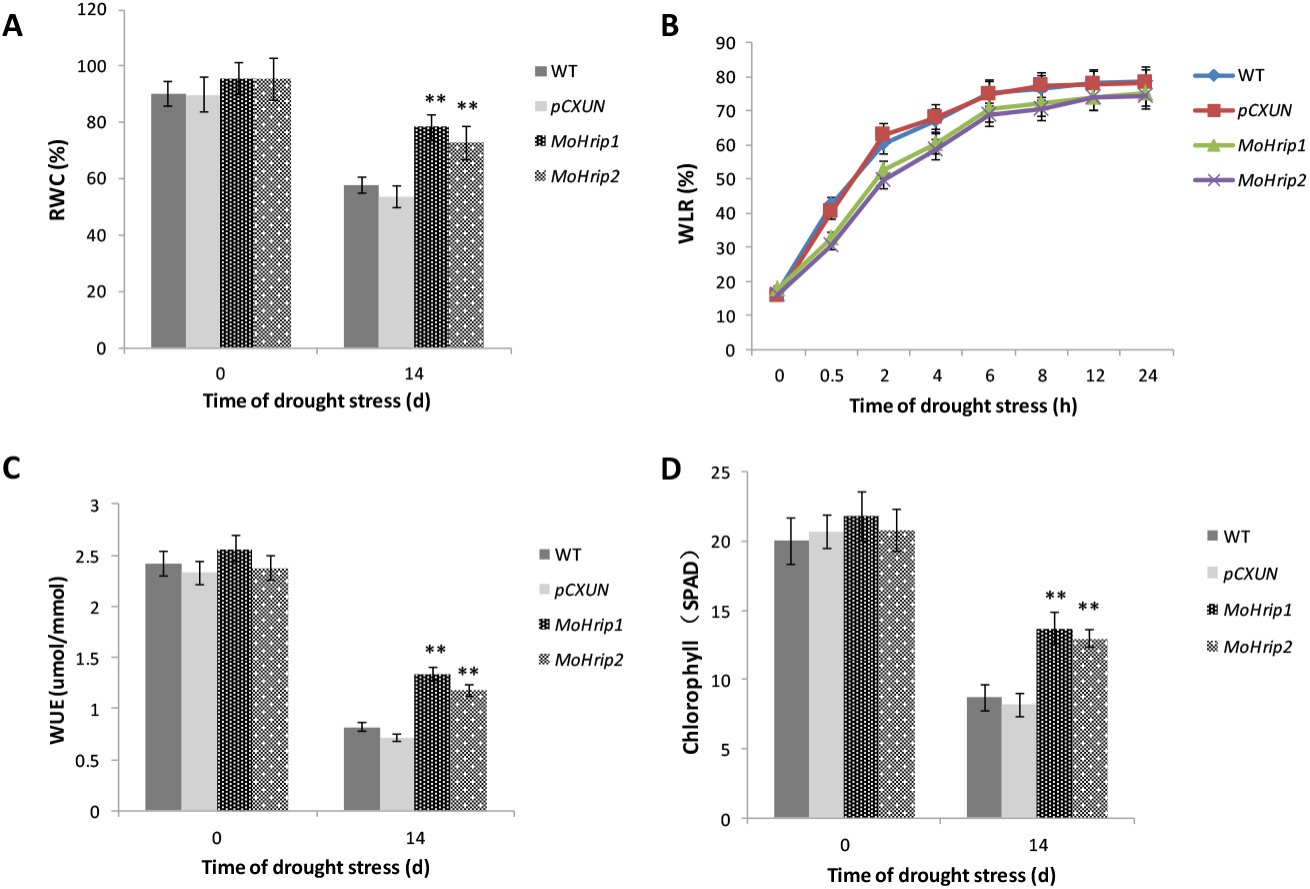
The physiological indices of drought-stressed rice. Analysis of RWC (A), WLR (B), WUE (C), and the chlorophyll content (D) in WT, *pCXUN* and transgenic rice under normal condition and after 14 d of drought stress. Error bars represent the mean ± SD of three replicates. Asterisks indicate significant differences from the WT rice (*P < 0.05, **P < 0.01).

There were no significant differences in the physiological indices between the control and transgenic rice plants under normal growth conditions, but clear differences were observed between the control and transgenic rice plants after drought treatment. Under drought stress, *MoHrip1* and *MoHrip2* plants retained a higher RWC and a lower WLR compared with those of WT and *pCXUN* plants (Fig 6A and 6B). These results indicated that MoHrip1 and MoHrip2 play a positive role in improving the ability of the transgenic plants to retain water under drought stress. During drought treatment, the difference in water usage efficiency (WUE) between the transgenic and the control rice was statistically significant (Fig 6C). The chlorophyll content was higher in *MoHrip1* and *MoHrip2* rice compared with the control rice after drought treatment (Fig 6D).

**Fig 7:**
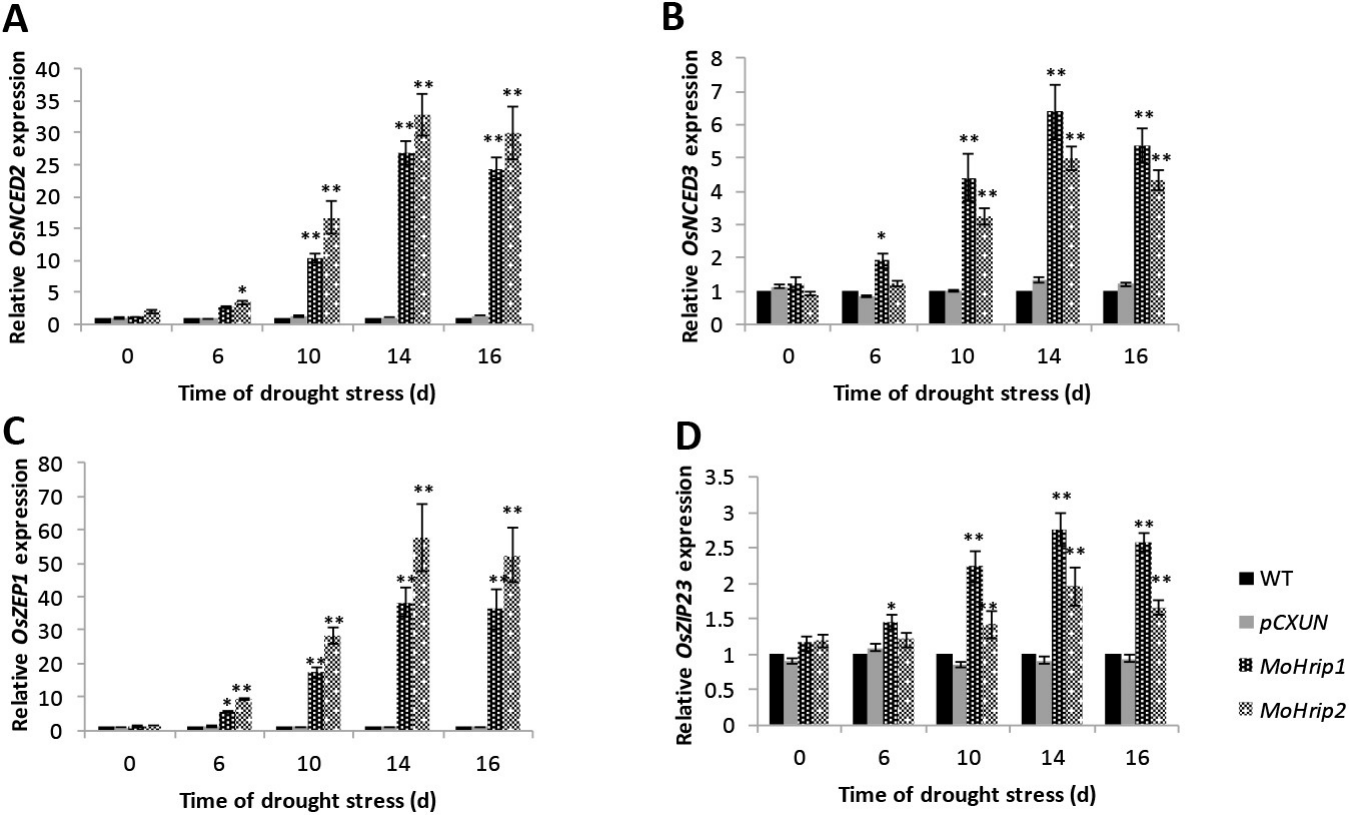
The expression levels of ABA-related genes in rice under drought-stress treatment. RNA samples were prepared from rice leaves collected on the 0 d, 6 d, 10 d, 14 d, and 16 d of drought stress. The relative expression of *OsNCED2* (A), *OsNCED3* (B), *OsZEP1* (C), and *OsbZIP23* (D) is shown. Error bars represent mean ± SD. Essentially identical results were obtained across three independent experiments. The asterisks indicate significant differences from the WT rice (*P < 0.05, **P < 0.01)

To further investigate the tolerance of the transgenic plants under drought stress, we analyzed the expression levels of the ABA biosynthesis-related genes *OsNCED2*, *OsNCED3*, and *OsZEP1* and the ABA signal-related gene *OsbZIP23* under drought-stress treatment. The transcript levels of all four genes were significantly up-regulated in the *MoHrip1* and *MoHrip2* rice plants. Compared with the control plants, the levels of these four genes were significantly increased in the *MoHrip1* and *MoHrip2* rice on day 10, 14, and 16 of drought stress (Fig 7).

### MoHrip1 or MoHrip2 expression promotes rice agronomic traits

*MoHrip1* and *MoHrip2* transgenic plants showed a faster germination rate and a longer bud and root length when compared with the WT and *pCXUN* rice (S1 Fig). The differences in the agronomic traits between the *pCXUN* rice and *MoHrip1* or *MoHrip2* rice were significant (Table 3). The expression of MoHrip1 or MoHrip2 significantly promoted plant height, thousand-kernel weight, tiller number, and ear number, and shortened the days of sowing to mowing.

**Table 3:** Improved agronomic traits in transgenic rice expressing MoHrip1 or MoHrip2.

Lines	Plant height/cm	Tiller number	Ear number	Thousand kernel weight/g	The days of sowing to mowing/day
***MoHrip1*::HA**	74.1±2.73 a	43.5±3.18 a	40.2±4.26 a	23.9±1.23 a	104.9±1.10 a
***MoHrip2*::HA**	63.6±2.36 b	40.5±4.06 a	38.2±5.63 a	21.7±0.830 a	104.4±1.27 a
***pCXUN***	52.3±3.14 c	23.0±7.48 b	18.0±5.85 b	16.1±0.532 b	114.6±1.25 b

Each line with 25 seedlings was performed in triplicate under the same environmental conditions. Different letters indicate significance at P<0.05.

## Discussion

Transgenic technology has become an efficient method to explore the function of exogenous genes in plants. MoHrip1 and MoHrip2 were isolated from the pathogenic fungus *M*. *oryzae*, and the gene sequences of the two elicitors were cloned [14,15]. In the present work, rice was successfully transformed with recombinant *mohrip1* and *mohrip2* independently. Then, the constitutive expression and stable inheritance of the genes in rice were used to determine the function of the two elicitors. *MoHrip1* and *MoHrip2* transgenic rice plants displayed enhanced resistance to *M*. *oryzae*, tolerance to drought stress and improved agronomic traits. The *mohrip1* and *mohrip2* gene regions without a signal peptide were fused with an HA-tag at the C-termini and expressed in transgenic rice. This tag did not affect the resistance of *MoHrip1* or *MoHrip2* rice in the present study; therefore, fusion proteins containing an HA-tag will be used for future research.

The protein elicitors produced by plant pathogens can enhance resistance to pathogen infection in transgenic plants [8–10,12,13]. Rice plants expressing MoHrip1 and MoHrip2 also presented enhanced resistance against *M*. *oryzae*. Different levels of pathogen resistance were observed among *MoHrip1*, *MoHrip2*, *pCXUN*, and WT rice. The disease scores suggested that *MoHrip1* and *MoHrip2* plants were more resistant to rice blast fungus compared with the control plants. The detached leaves of *MoHrip1* and *MoHrip2* plants showed small and constrained lesions, whereas the control plants showed severe lesions at 7 dpi.

Trypan blue staining was performed to explore the development of disease symptoms by observing hyphal morphology [2,27]. *MoHrip1* and *MoHrip2* rice showed coiled and constricted hyphae, suggesting that MoHrip1 and MoHrip2 inhibit the growth of hyphae in rice. Similar results have been reported previously [2,12]. Slightly fewer *M*. *oryzae* hyphae were observed within the *MoHrip1* rice than in the *MoHrip2* rice which also suggests that MoHrip1 is slightly stronger than MoHrip2 with regard to the inhibition of hyphal growth against rice blast fungus. Although the function of MoHrip1 or MoHrip2 in *M*. *oryzae* has not been fully elucidated, the two elicitors exhibited enhanced resistance to this pathogen in rice.

The SA pathway is the major signaling pathway induced by MoHrip1 and MoHrip2 proteins during rice blast resistance. Plants that encounter pathogen infection activate a defense system that involves two major pathogen defense signaling pathways: the SA-dependent pathway and the JA/Et-dependent pathway [28]. Pathogen-related (PR) proteins are involved in the plant defense network [29]. The expression of PR genes and the production of disease resistance substances are related to the SA pathway [30]. *PR-1a* and *PR-10a* were up-regulated in *MoHrip1* and *MoHrip2* rice according to qRT-PCR. The *PAL1* gene, which encodes the key enzyme for SA biosynthesis in the SA pathway, was clearly expressed in transgenic rice. The *EDS1* and *NH1* genes, which function downstream of the SA signaling pathway [31,32], were expressed at higher levels in the *MoHrip1* and *MoHrip2* rice than in the control rice. The *LOX2* and *AOS2* genes encode two important enzymes in the JA pathway [33], but *OsAOS2* was expressed in different pattern between them. These results suggest that the MoHrip1 or MoHrip2 expression in rice primarily triggers the SA-dependent pathway when a pathogen-plant interaction occurs.

Previous work revealed that expression of the harpin-encoding gene *hrf1* in rice increased the ABA content and water retention ability and enhanced drought tolerance [34]. In the present study, the enhance tolerance to drought stress of MoHrip1- and MoHrip2-expressing rice might be related to the regulation of ABA biosynthesis and the capacity for water retention. The phytohormones ABA control various aspects of plant growth and development [35,36]. Under drought stress, increases in endogenous ABA in seedlings help plants adjust to adverse situations [37]. After drought, the ABA content was higher in the *MoHrip1* and *MoHrip2* rice than that in the control rice, which indicate that the transgenic rice showed more drought tolerance.

During the response and adaptation to abiotic stress, many stress-related genes are activated. Most reports have shown that ABA biosynthetic pathway genes are up- or down-regulated in response to drought stress [38,39]. The ABA biosynthetic-related genes *OsNCED2*, *OsNCED3*, and *OsZEP1* encode the first two enzymes of the ABA biosynthetic pathway, zeaxanthin epoxidase (ZEP) and 9-cis-epoxycarotenoid dioxygenase (NCED), and these enzymes are significantly induced under stress conditions [40,41]. *OsbZIP23* belongs to the basic leucine zipper (*bZIP*) family, whose members play a role in plant stress-responsive and hormone signal transduction [42,43]. We found that the expression levels of *OsNCED2*, *OsNCED3*, *OsZEP1*, and *OsbZIP23* increased in the *MoHrip1* and *MoHrip2* rice compared with control rice. These results suggest that MoHrip1 and MoHrip2 contribute to the enhanced drought-stress tolerance of transgenic rice through the elevated expression of key genes. Similar results have been reported previously [44]. The enhanced drought tolerance of MoHrip1- and MoHrip2-expressing transgenic rice might be related to the ABA-biosynthesis pathway.

Under drought stress, *MoHrip1* and *MoHrip2* rice, which displayed a higher capacity for water retention, survived better compared with controls. A high water-retention ability allows plants to stay green to maintain the crop canopy and help crops endure drought conditions [45]. The higher RWC, WUE and a lower WLR of transgenic rice indicated that MoHrip1and MoHrip2 expression in rice might be attributed to the capacity for water retention in drought stress.

An agricultural trait study was conducted comparing the control with the transgenic plants in addition to the comparison of growth performances under normal conditions. MoHrip1 and MoHrip2 expression in rice resulted in a significant increment with respect to the germination rate, plant height, and yield of rice. This implied that *MoHrip1*and *MoHrip2* transgenic rice could grow well, which may enhance their ability to withstand adverse environments.

Both *MoHrip1* and *MoHrip2* transgenic plants show enhanced resistance to rice blast and drought stress. MoHrip1 and MoHrip2 function similarly in rice, and this similarity might be related to the structures of the two elicitors. The three-dimensional structure of a protein is closely related to its biological function. Crystallization and preliminary X-ray diffraction analyses of MoHrip1 and MoHrip2 have been reported previously [46,47]. The crystal structures of MoHrip1 was recently determined (Zhang, personal communication), and the crystal structures of MoHrip2 was reported recently [48]. Although MoHrip1 showed a low similarity with MoHrip2 in terms of its primary structure, both had similar three-dimensional structures composed of β-barrels.

In summary, the knowledge that MoHrip1 and MoHrip2 enhance disease resistance and drought tolerance might lead to a feasible approach to obtain stable and high rice yields in the future. The relationships among disease resistance, drought tolerance and plant hormone levels warrant future research. The results described here might provide the theoretical and technological basis for understanding the resistance mechanisms of transgenic rice.

## Supporting information

S1 Table Sequences of primers for qRT-PCR. (DOCX)

S2 Table The results of resistance selection to T_1_ generation transgenic rice. (DOCX)

S1 Fig The germination rates, bud and root length were measured. The germination rates of the *MoHrip1*, *MoHrip2*, *pCXUN*, and WT rice seeds (A). The bud and root length of the *MoHrip1*, *MoHrip2*, *pCXUN*, and WT rice (B, C).
(PDF)
