# Supplementary material for: Enhanced disease resistance and drought tolerance in transgenic rice plants overexpressing protein elicitors from Magnaporthe oryzae
Source: PLoS One. 2017 Apr 18;12(4):e0175734. doi: 10.1371/journal.pone.0175734 (PMC5395183; doi:10.1371/journal.pone.0175734)
Supplement: S1 Table — (DOCX) [file pone.0175734.s001.docx]

**S1 Table**

Sequences of primers for qRT-PCR.

| Gene | Accession numbers | Forward primer(5’-3’) | Reverse primer(5’-3’) |
| --- | --- | --- | --- |
| *Osactin* | AK060893 | GAGTATGATGAGTCGGGTCCAG | GAGTATGATGAGTCGGGTCCAG |
| *OsPR-1a* | AJ278436 | GTCGGAGAAGCAGTGGTA | CGAGTAGTTGCAGGTGATG |
| *OsPR-10a* | AF274850 | GGCTTGGTCGACGACATTG | CAGGGTTAAGCTTCATGGTGTAGA |
| *OsEDS1* | AK100117 | CCCCGCATACCACTTACT | TGTTGATGAAACCACTCCC |
| *OsPAL1* | AK068993 | GGTGTTCTGCGAGGTGATGA | AGGGTGGTGCTTCAGCTTGT |
| *OsNH1* | AK120715 | ATCTTGATGATGCGTTTGC | TCAGCTTGCTCCAGTATTTC |
| *OsLOX2* | AK241395 | AGATGAGGCGCGTGATGAC | CATGGAAGTCGAGCATGAACA |
| *OsAOS2* | AK061758 | TACCAGCCGTGCGCCACCAG | AGGACGGAGCTGGTTGAGTGG |
| *OsNCED2* | AY838898 | GGAGAGAGTTGGTTTGTG | ATTGTTGTGCGAGAAGTT |
| *OsNCED3* | AY838899 | CAGGATATGCTCACATACAG | GGAGAATCTCACCGAATTG |
| *OsZEP1* | LOC_Os04g37619 | GAAGTCTAATGATACGGAATCT | TGGTTCTCAAGTGTCTCA |
| *OsbZIP23* | KP779640 | CCAGAGGAAACAGGCATAT | ACTTGTCGGCTCATTCTC |
