# Supplementary material for: Enhanced disease resistance and drought tolerance in transgenic rice plants overexpressing protein elicitors from Magnaporthe oryzae
Source: PLoS One. 2017 Apr 18;12(4):e0175734. doi: 10.1371/journal.pone.0175734 (PMC5395183; doi:10.1371/journal.pone.0175734)
Supplement: S2 Table — (DOCX) [file pone.0175734.s002.docx]

**S2 Table**

The results of resistance selection to T_1_ generation transgenic rice

| Lines | Seed numbers | Germination | Non germination | Rate |
| --- | --- | --- | --- | --- |
| *MoHrip1*-2 | 40 | 28 | 12 | 2.3:1 |
| *MoHrip1*::HA-19 | 30 | 23 | 7 | 3.3:1 |
| *MoHrip2*-2 | 50 | 35 | 15 | 2.3:1 |
| *MoHrip2*::HA-3 | 50 | 37 | 13 | 2.8:1 |

Different lines of transgenic rice plants were chosen for resistive selection. The rice seeds were placed in a 1/2 MS liquid culture medium (100 mg L^-1^ hygromycin) for 24 h to germinate, and then 15-days seedlings underwent PCR detection to determine positive lines. The transgenic plants were segregated at 3:1 for hygromycin resistance.
