# Supplementary material for: Enhanced disease resistance and drought tolerance in transgenic rice plants overexpressing protein elicitors from Magnaporthe oryzae
Source: PLoS One. 2017 Apr 18;12(4):e0175734. doi: 10.1371/journal.pone.0175734 (PMC5395183; doi:10.1371/journal.pone.0175734)
Supplement: S1 Fig — The germination rates of the MoHrip1, MoHrip2, pCXUN, and WT rice seeds (A). The bud and root length of the MoHrip1, MoHrip2, pCXUN, and WT rice (B, C). (PDF) [file pone.0175734.s003.pdf]

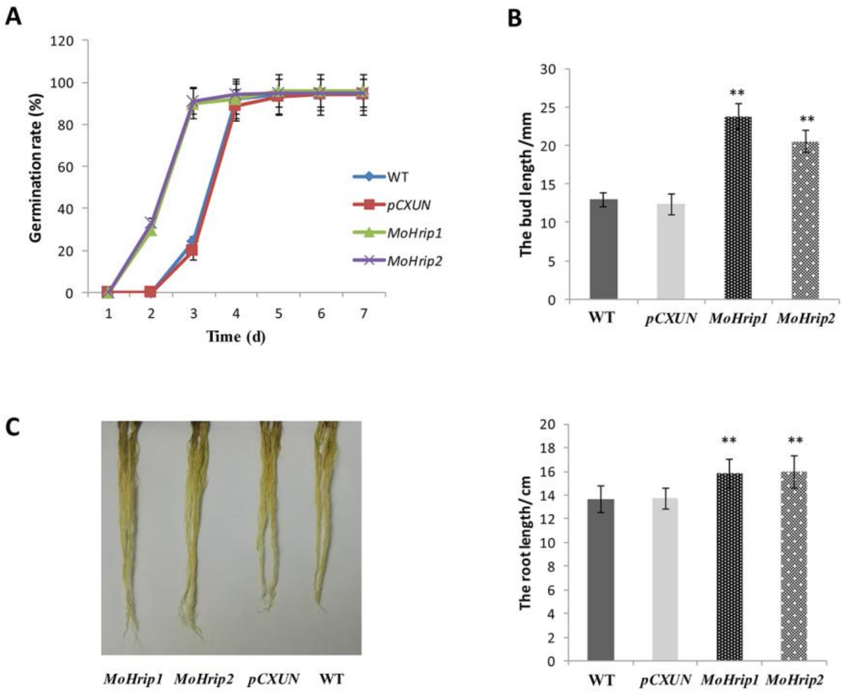

S1 Fig. The germination rates, bud and root length were measured. The germination rates of the *MoHrip1*, *MoHrip2*, *pCXUN*, and WT rice seeds (A). The bud and root length of the *MoHrip1*, *MoHrip2*, *pCXUN*, and WT rice (B, C).
